# Eeg Coherence between Prefrontal and Posterior Cortical Regions is Related to Negative Personality Traits

**DOI:** 10.3389/fnhum.2012.00269

**Published:** 2012-10-04

**Authors:** Linda Isaac, Peter J. Bayley

**Affiliations:** ^1^The Veterans Affairs Palo Alto HealthCare System, War Related Illness and Injury Study CenterPalo Alto, CA, USA; ^2^Department of Psychiatry and Behavioral Sciences, Stanford University School of MedicineStanford, CA, USA

Despite the high prevalence of mood disorders, their underlying psychopathology remains poorly understood. A recent publication by Reiser et al. (2012) in *Brain and Cognition* describes a possible mechanism underlying the development and maintenance of attentional bias in depression and anxiety. Their findings regarding decoupling of prefrontal–posterior brain regions adds to the existing evidence that fronto-limbic interactions facilitate the generation of task-relevant responses while inhibiting interference from emotionally distracting information. The authors employed electroencephalogram (EEG) and three mood induction methods (sad, anxious, and neutral moods) in healthy controls to explore state-dependent coherence between prefrontal and posterior cortical regions as a mechanism for modulating the impact that social-emotional information has on an individual. The findings suggest that prefrontal–posterior decoupling is related to individual differences in the behavioral traits of absorption and in the propensity to ruminate. Higher scores in these traits were related to loosened coupling between prefrontal cortex (PFC) and posterior cortex. Conversely, healthy controls with lower scores on absorption and rumination showed stronger prefrontal–posterior coupling. Results were interpreted as showing that less prefrontal–posterior coupling may be related to loosening of control by the PFC over incoming social-emotional information, and consequently to deeper emotional involvement and absorption. Conversely, increased prefrontal–posterior coupling may be related to strong control, with a consequent dampening of emotional experience and a lack of emotional engagement. When considering the neural substrates of emotional control, the limbic system has been suggested to play a key role. This set of brain regions includes frontal and medial temporal lobe structures. Although there is no universally accepted definition of what constitutes the limbic system, the anterior cingulate cortex (ACC) is thought to play an important role in emotional regulation. Numerous other functions have been ascribed to the ACC but here we focus on the specific role of the ACC on regulating emotion through attentional control.

The ACC can be divided into dorsal (cognitive), and ventral (emotional) components. The dorsal ACC is thought to monitor for errors or processing conflicts that could disrupt performance and to recruit the dorsolateral PFC (DLPFC) to reallocate attentional resources as needed (Allman, et al., 2001). Specifically, cognitive reallocation by the ACC is thought to be activated by demanding tasks that involve stimulus-response selection in the face of competing streams of information such as emotionally valenced distractions. The authors did not include a task that forced the engagement of this reallocation system to better account for the relationship between mood and inhibitory deficits. This could have been accomplished for example by employing a classic EEG paradigm (Go/NoGo). Moreover, while methods such as high-density EEG, independent component analysis and surface Laplacian estimation afford the opportunity for greater spatial resolution of cognitive tasks, it is the temporal precision of EEG compared to other imaging methods which leads to its superiority in such experiments. As a result, this technique could have been utilized to understand “when” (not just where) the decoupling occurs and whether time-course differences could provide additional explanatory power to differentiate between individual differences in absorption or rumination. Similar questions could also be asked of patient groups. For example, does decreased prefrontal–posterior EEG coherence in patients with anxiety disorders occur earlier (vigilant orientation) compared to those with depression (disengagement deficits or perseverative errors) during affective challenges? A neurophysiological index of prefrontal inhibitory control was detected with a negative-going deflection peaking between 250 and 350  ms after the onset of NoGo distractors. This phasic negativity often peaks in midfrontal scalp regions and has been considered to be the electrophysiological correlate of ACC function involved in sustained attention, error detection, and response control. Thus, the N2 component of the event-related potential, an evoked response presumably generated within the ACC, is significantly enhanced when individuals successfully inhibit a response, and therefore provides an excellent neurophysiologic metric of frontal inhibitory function (Nieuwenhuis et al., 2003).

Importantly, recent research has demonstrated inhibitory dysfunction in depression and anxiety, and this deficit is likely to be valence-specific. It is often referred to as *attentional bias*, broadly defined as the preferential processing of mood-congruent stimuli over neutral stimuli. Attentional bias is perhaps the clearest example of when the ACC-DLPFC relationship is unsuccessful through a failure to ward off emotional distracters. We propose that stimulus control is essential in defining models of inhibitory deficits for both depression and anxiety. For instance, one can imagine there may be differences in prefrontal–posterior EEG coherence when comparing an angry face with an averted vs. a direct gaze because the latter signals impending threat toward the observer. We also argue that the process of experimental mood induction is not always straightforward. For instance, observing a sad person does not necessarily induce a sad mood in a participant as this bears no direct relevance or importance to them. In fact, an angry face may be more salient to a sad or depressed observer than a control participant due to the message of rejection or disapproval (Isaac et al., 2012). The authors accordingly note that mood induction is transient, and, as we have found in our own research, manipulation checks in mood induction designs are especially important because moods can occur concomitantly (sad and anxious) or change from sad to anxious during the experiment. Interestingly, the authors also asked participants in the three mood states to rate additional emotions, including disgust. This more complex emotion recruits different brain regions such as the orbito-frontal and insular cortices commonly affected in neurological disorders. Specifically, disgust has been observed to be compromised in neurodegenerative diseases, such as the behavioral variant of fronto-temporal dementia (Sturm et al., 2006), rather than affective disorders.

Excitingly, the decoupling hypothesis, sometimes referred to as “hypofrontality,” is applicable to a host of psychological disorders apart from depression and anxiety, and thus holds high explanatory value for what could be a common causal mechanism for the development and maintenance of psychopathology in general. For instance, several strands of functional neuroimaging evidence have shown PFC dysfunction in schizophrenia, with specific evidence for reduced attentional control relative to controls during emotional distraction and deactivation failure during cognitive tasks (Dichter et al., 2010). In depressed subjects, increased limbic activity has been shown in response to emotional stimuli, and decreased DLPFC activity during cognitive tasks (Siegle et al., 2007). This may indicate an inability to effectively recruit the DLPFC, leading to a failure to inhibit limbic activity, especially in the presence of task-irrelevant and novel valenced stimuli. Interestingly, lower DLPFC and ACC activation levels also characterize high-risk groups, such as formerly depressed participants (Hooley et al., 2009), suggesting that an altered cortico-limbic relationship is not necessarily reversed by successful treatment of such disorders. This observation has ignited debates about whether hypofrontality is a cause or consequence of disease and if disease can be ameliorated by identifying hypofrontality in subclinical groups (e.g., dysthymic patients). Future studies are required to determine whether alterations in frontal activation correlate with disease symptoms and therefore constitute a risk factor for psychiatric illness. Importantly, the study by Hooley et al. (2009) suggests that self-report measures and clinical status are not sufficient for detecting what may be subtle deficiencies in the reciprocal relationship between the DLPFC and ACC. It argues for the use of neuroimaging techniques that are sensitive enough to detect such subtleties.

The elucidation of the neural circuitry underlying the control of emotional experience contributes significantly to our comprehension of the symptoms of depression and anxiety, such as rumination and hypervigilance, respectively. For example, depressed patients may succeed at conflict detection in the form of hypersensitivity to negative cues evidenced by the engagement of a key emotional processing structure (amygdala) but fail on tasks requiring conflict resolution that involve cognitive control (DLPFC-ACC). We suggest that closing the loop between the emotional and the cognitive brain is central to progress in the treatment of emotional disorders. These reciprocal regulatory mechanisms constitute a critical property of human adaption that facilitates the balance between processing task-relevant information and emotionally salient but task-irrelevant information. Successful goal directed behavior appears to be mediated by direct feedback connections between the DLPFC and the ACC especially when emotional distracters are present.
